# Supplementary material for: Polyamine Metabolism under Different Light Regimes in Wheat
Source: Int J Mol Sci. 2021 Oct 29;22(21):11717. doi: 10.3390/ijms222111717 (PMC8583935; doi:10.3390/ijms222111717)
Supplement: Supplementary file 1 [file ijms-22-11717-s001.zip › Supplementary Table S2.pdf]

Supplementary Table S2. Carotenoid UPLC-PDA-MS method details

**UPLC:**	**Waters Acquity I-class**		
**Column:**	Thermo Accucore C30 2.6 µm 4.6x150 mm, thermostated at 8°C (to enhance geometric isomer separation)		
**inj vol.:**		10 µl	
**Autosampler temperature:**		6 °C	
**Gradient conditions:**		**A: MeOH:H2O:tert-butyl methyl ether (TBME) 70:30:30 v/v%**	**B: MeOH:TBME 50:50 v/v%**
Time (min)	Flow rate [illegible]	A%	B%
0	0.65	75	25
2	0.65	70	30
12	0.75	55	45
24	1	0	100
26	1	0	100
27	0.75	75	25
28	0.65	75	25
32	0.65	75	25
**Detector 1:**	**PDA - 250-700 nm; 20 scans/sec, 1.2nm resolution**		
**Detector 2:**	**Xevo TQ-XS with Unispray source**		
**Resolution:**	Unit mass (+/- 0.8 Da)		
**Impactor voltage:**		2 kV	
**Polarity:**		UniSpray Positive	
**Desolvation temperature:**		450°C	
**Nebulizer gas:**		6.5 bar N_2	
**Desolvation gas flow:**		1000 L/h N_2	
**Cone gas flow:**		450 L/h N_2	
Collision gas flow:		0.15 ml/min Argon 5.0	
Collision energy:		4 eV	
**Mode:**		MS-scan, 280-2000 m/z	
**Components evaluated in samples:**			
Quant trace (nm)	Retention time (min)	name	
440	5.0	*trans*-violaxanthin	
436	5.2	*trans*-neoxhantin	
445	8.8	trans-lutein	
465	14.4	chlorophyll *b*	
430	17.2	chlorophyll *a*	
409	21.1	pheophytin *a*	
450	22.4	β-carotene	
450	22.8	9-*cis*-β-carotene	
