# Supplementary material for: Polyamine Metabolism under Different Light Regimes in Wheat
Source: Int J Mol Sci. 2021 Oct 29;22(21):11717. doi: 10.3390/ijms222111717 (PMC8583935; doi:10.3390/ijms222111717)
Supplement: Supplementary file 1 [file ijms-22-11717-s001.zip › Supplementary Table S3.pdf]

Supplementary Table S3. Gene-specific and housekeeping primers.

Gene name	Primer sequences (5' → 3')		Amplicon size	Reference
TaPAL	Forward	CCATCACCAAGCTGCTCAAC	106 bp	AY005474.1
	Reverse	ATAAGGCCGGCAATGTAGG		
TaperPAO	Forward	GCTCATAAATCAGCCCAATTCCA	113 bp	[90]
	Reverse	TTCGCCATTTGTTGAGCTCT		
TaSPDS	Forward	AGGTATTCAAGGGTGGCGTG	125 bp	[12]
	Reverse	TGGGTTCACAGGAGTCAGGA		
TaADC	Forward	TCTACCCCGTCAAGTGCAAC	128 bp	[12]
	Reverse	GACGAGGCAGCTCATGGT		
TaCS	Forward	GCGGCCATCGTCTCCACCAT	192 bp	[91]
	Reverse	GGCCGAGGTACAGGGAGGGA		
TaICS	Forward	TTCAGCTCCACCAAACCAACCA	99 bp	[91]
	Reverse	GGTTTGCCCACTGAAGAAGCG		
TaNCED	Forward	CCTCGAAGCCCAGCACTAAT	74 bp	[92]
	Reverse	GAGAGCGAGAGGTCCAATGG		
TaSAMDC	Forward	ACAGCCTTCTCCACACAAGA	195 bp	[12]
	Reverse	TCCAGACCAGTCATGCACA		
TaODC	Forward	GATATCGGCGGCGGGTTCAT	109 bp	HM770451.1
	Reverse	CTATCACCTCCACGCACGGA		
TaPAO	Forward	CCAGCCTCCAGCTCCGCAAC	137 bp	[90]
	Reverse	GCCCAGCTCCTCCACCTCGTC		
Ta2291	Forward	GCTCTCCAACAACATTGCCAAC	165 bp	[93]
	Reverse	GCTTCTGCCTGTCACATACGC		
