# Supplementary figures and images for: Polyamine Metabolism under Different Light Regimes in Wheat
Source: Int J Mol Sci. 2021 Oct 29;22(21):11717. doi: 10.3390/ijms222111717 (PMC8583935; doi:10.3390/ijms222111717)

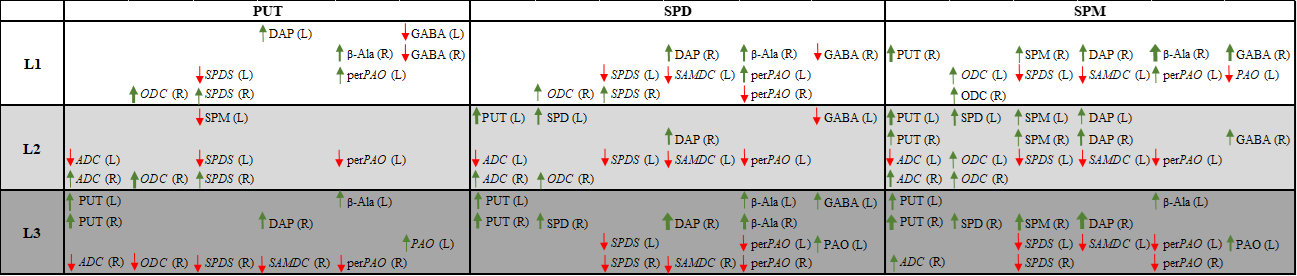

Supplement: Supplementary file 1 [file ijms-22-11717-s001.zip › Supplementary Table S1.tif]
